# CEBPB/POU2F2 modulates endothelin 1 expression in prehypertensive SHR vascular smooth muscle cells

**DOI:** 10.1530/JME-22-0178

**Published:** 2023-05-02

**Authors:** Tien-Chun Yang, Mei-Hua Lu, Wei-Jie Wang, Jang-Yi Chen

**Affiliations:** 1 Department of Anatomy and Cell Biology, School of Medicine, College of Medicine, Taipei Medical University, Taipei, Taiwan; 2 Department of Biology and Anatomy, National Defense Medical Center, Taipei, Taiwan; 3 Department of Entomology, University of California, Riverside, California, USA

**Keywords:** hypertension, spontaneously hypertensive rats, endothelin 1, aorta, vascular smooth muscle cell

## Abstract

The pathogenesis of hypertension is not fully understood; endothelin 1 (EDN1) is involved in developing essential hypertension. EDN1 can promote vascular smooth muscle cell (VSMC) proliferation or hypertrophy through autocrine and paracrine effects. Proliferating smooth muscle cells in the aorta are 'dedifferentiated' cells that cause increased arterial stiffness and remodeling. Male SHRs had higher aortic stiffness than normal control male WKY rats. Male SHR VSMCs expressed high levels of the *EDN1* gene, but endothelial cells did not. Therefore, it is necessary to understand the molecular mechanism of enhanced EDN1 expression in SHR VSMCs. We identified POU2F2 and CEBPB as the main molecules that enhance EDN1 expression in male SHR VSMCs. A promoter activity analysis confirmed that the enhancer region of the *Edn1* promoter in male SHR VSMCs was from −1309 to −1279 bp. POU2F2 and CEBPB exhibited an additive role in the enhancer region of the *EdnET1* promoter. POU2F2 or CEBPB overexpression sufficiently increased EDN1 expression, and co-transfection with the CEBPB and POU2F2 expression plasmids had additive effects on the activity of the *Edn1* promoter and EDN1 secretion level of male WKY VSMCs. In addition, the knockdown of POU2F2 also revealed that POU2F2 is necessary to enhance EDN1 expression in SHR VSMCs. The enhancer region of the *Edn1* promoter is highly conserved in rats, mice, and humans. POU2F2 and CEBPB mRNA levels were significantly increased in remodeled human VMSCs. In conclusion, the novel regulation of POU2F2 and CEBPB in VSMCs will help us understand the pathogenesis of hypertension and support the development of future treatments for hypertension.

## Introduction

During the development of hypertension, vascular remodeling leads to increased vessel wall thickness, decreased lumen diameter, and increased blood pressure (BP) (Roman *et al.* 1992). Vascular remodeling involves hypertrophy and dedifferentiation of vascular smooth muscle cells (VSMCs) (Brown *et al.* 2018, Touyz *et al.* 2018). These transitions of VSMCs in the aorta lead to increased medial wall thickness and wall stiffness, which are also essential components of increased BP (Sehgel *et al.* 2013, Laurent & Boutouyrie 2015). Spontaneously hypertensive rats (SHRs) are inbred rats whose BP gradually increases after growing to puberty with a natural diet and rearing environment (Hamada *et al.* 1990). In SHRs, the vascular wall thickness increases proportionally to the elevation in systolic BP (Olivetti *et al.* 1982). The growth rate of SHR VSMCs at the hypertensive stage was higher than that of VSMCs in normotensive rats (Scott-Burden *et al.* 1989, Hamada *et al.* 1990). Therefore, the decrease in vessel diameter and increase in vessel stiffness caused by VSMC remodeling are integral parts of the development of hypertension. Various vasoactive factors, including endothelin 1 (EDN1), thromboxane A2, and the vasodilator, prostacyclin, contribute to the growth of VSMCs (Hirata *et al.* 1989, Peiró *et al.* 1995, Lu *et al.* 2003). In particular, EDN1 regulates smooth muscle cell constriction (SMC) and structural remodeling in paracrine and autocrine manners (Hahn *et al.* 1990).

EDN1 is a 21-residue peptide vasoconstrictor originally isolated from endothelial cells (ECs) supernatants (Yanagisawa *et al.* 1988). In addition, EDN1 was also found in VSMCs (Woods *et al.* 1999), cardiomyocytes (Suzuki *et al.* 1993), and alveolar ECs (Markewitz *et al.* 1995). Aberrant expression of EDN1 affects atherosclerosis and some induced hypertensive animals (Schiffrin *et al.* 2001, Schmitz-Spanke & Schipke 2000). EDN1 is involved in different developmental stages of hypertension. In the early stage, EDN1 upregulates the nicotinamide adenine dinucleotide phosphate oxidase expression level in vascular ECs and promotes the production of reactive oxygen species (Mohazzab *et al.* 1994, Touyz *et al.* 2020). In addition, EDN1 also induces macrophages to release inflammatory factors such as tumor necrosis factor-alpha, interleukin (IL) 1, IL6, and IL8 (Ruetten & Thiemermann 1997, Hofman *et al.* 1998, Browatzki *et al.* 2000, Yang *et al.* 2004). In the mid-term, EDN1 induces transitions in VSMC morphology and function, and the phenotype of cells shifts to a synthetic phenotype with proliferative and migratory abilities. Thus, ET1 increases vessel stiffness and diameters (Iglarz & Schiffrin 2003, Amiri *et al.* 2004). Finally, EDN1 induces upregulation of renal tubular reabsorption of water and electrolytes in the kidneys which increases the blood volume and reduces peripheral vessel diameters to decrease the vascular volume (Kostov *et al.* 2021).

Among young people, men have a higher rate of high BP than women, and after menopause, women tend to have higher BP values than men (Ramirez & Sullivan 2018). BP values in SHR showed similar sex differences. BP was significantly higher in 4- to 8-month-old male SHR than in females, but the opposite was true in 18-month-old rats (Reckelhoff & Fortepiani 2004). This result indicates that estrogen has an important influence on BP regulation. Clinical studies have reported higher plasma EDN1 levels in men than in women (Tostes *et al.* 2008). The sex difference in EDN1 regulation of BP may be due to the expression of ETA receptor in the kidneys of men is higher than women (Intapad *et al.* 2015). Our previous study showed that the level of EDN1 secreted by VSMCs in male SHRs was 2.5-fold higher than in male WKY rats (Lu *et al.* 2001, 2003). We also found that the proliferation of SHR VSMCs was mainly due to an autocrine effect of EDN1 (Lu *et al.* 2006). Those findings suggested that higher levels of EDN1 in male SHR VSMCs influence the development of hypertension. In mammalian ECs, a 150-bp upstream region of the transcription start site contains several highly conserved transcription factor-binding motifs, such as a thymine-adenine-thymine-adenine (TATA) box, a vascular endothelial zinc finger 1-binding site, a FOXO binding site, a CAAT box, an activating protein 1 (AP-1)-binding site, a hypoxia-inducible factor-1-binding site, and a GATA box (Stow *et al.* 2011). These elements located in the proximal promoter region can indirectly or directly recruit RNA polymerase II and regulate the basal transcriptional activity of the gene (Lee *et al.* 1990). Previously studies reported that CCAAT/enhancer-binding protein-beta (CEBPB) gene expression levels were 1.25-fold upregulated in prehypertensive SHRs and 1.52-fold upregulated in BP high (BPH) mice (Friese *et al.* 2005). CEBPB also increased ET1 expression in hypercholesterolemic rabbits and modulated balloon injury-induced vascular lesion formation (Kelkenberg *et al.* 2002). Lauth and colleagues also demonstrate that high BP modulates ET1 gene expression through CEBPB and CCAAT/enhancer-binding protein-delta in porcine and human ECs (Lauth *et al.* 2000). In this study, we provide evidence to support the hypothesis that POU class 2 homeobox 2 (POU2F2) and CEBPB upregulation is involved in the EDN1 overexpression of SHR VSMCs. POU2F2 and CEBPB upregulation-induced EDN1 overexpression can be accounted for by an additive effect of POU2F2- and CEBPB-induced promoter activities and targeting of the *Edn1* promoter enhancer region. Elucidating the novel role of POU2F2 and CEBPB can assist our understanding of the pathogenesis of hypertension and supports the development of future treatments for hypertension.

## Materials and methods

### Animals

Three- to four-week-old male SHR and WKY rats in pathogen-free cages are handled according to the National Research Council's Guide for the Care and Use of Laboratory Animals. All anesthesia and sacrifice procedures were reviewed and approved by the Institutional Animal Care and Use Committee of the National Defense Medical Center (NDMC; IACUC-05-158). Rats were anesthetized with 50 mg/kg pentobarbital (Sigma-Aldrich) by intraperitoneal injection and placed under a dissecting microscope (SZX16, Olympus, Japan)

### Cell culture

The rat VSMC line (A10) was obtained from the Bioresource Collection and Research Center (Hsinchu, Taiwan). Primary aortic SMCs of SHRs and WKY rats were isolated and cultured (until passage 3–5) as previously described (Scott-Burden *et al.* 1989). We isolated aortas from three rats for each VSMC culture from SHR or WKY rats. All cells were cultured in Dulbecco’s modified Eagle’s medium (DMEM; Hyclone, Logan, UT, USA) supplemented with 10% fetal bovine serum (Hyclone) at 37°C and 5% CO_2_.

### Plasmids

The rat *Edn1* promoter construct of the 1309prET1 plasmid was kindly provided by Dr Martin Paul (Free University, Germany) (Paul *et al.* 1995). The 1309prET1 plasmid was inserted into pGL3-enhancer with firefly luciferase reporter gene vector (Promega) at the XhoI-HindIII cloning site. Designed deletion mutants of different length fragments of the ET1 promoter were generated using primers 1279prET1 (sense, 5’-AGGAGCTCTGTCACTTGTACCTTAATAAC-3’), 1143prET1 (sense, 5’-CTCGAGCTCGAGTCAGCATAGGCAGTC-3’), 760prET1 (sense, 5’-CTCCTCTCGAGGCACAGGGAATTTTG-3’), 557prET1 (sense, 5’-CTCCTCTCGAGGGGAGTTTGGGAAAAG-3’), 81prET1 (sense, 5’-CTCCTCTCGGACGGCTGGAATAAAG-3’), and reverse primer    (5’-AAGCTTAAGCTTCAGCGCGGTCTTCAAAAAG-3’).

The CEBPB-binding motif and POU-specific (POUs) domain-binding motif mutant constructs were subjected to site-directed mutagenesis using a polymerase chain reaction (PCR) with the forward primer (CEBPB-binding motif mutation: TGTGTTTCCATTTATTCATGAAGACATGTT; POUs domain-binding motif mutation: TGTGTTTTGCTTTATTTGCGAAGACATGTT; CEBPB POUs double mutation: TGTGTTTCCATTTATTTGCGAAGACATGTT), and reverse primer (AACACCAGGGGGAGACGAAG). Mutated nucleotides in the reported sequences are underlined. The 1309prET1ΔC plasmid contained the site-directed mutant of CEBPB. The 1309prET1ΔP plasmid contained the site-directed mutant of the POUs-binding site of the GHF-1 consensus core sequence. The 1309prET1ΔCP plasmid contained the site-directed mutant of CEBPB and the POUs-binding sequence.

The CMV-based expression vector encoding *Cebpb* was kindly provided by Dr Sheng-Chung Lee (National Taiwan University, Taiwan) (Su *et al.* 2003). The human *POU2F2* gene expression vector was kindly provided by Dr Jiann-Shiun Lai (Cold Spring Harbor, USA) (Lai *et al.* 1992). The *POU2F2* and CEBPB genes were subcloned into the NotI-XhoI site of the MYC-his tag containing the pcDNA3.1A vector (Invitrogen).

The TRCN0000081519 (sh*PoU2f2*-1) clone, TRCN0000081522 (sh*Pou2f2*-2) clone, pLKO.1-shLuc vector (control), pMD.G plasmid, and pCMVΔR8.91 plasmid were obtained from the National RNAi Core Facility at the Institute of Molecular Biology, Academia Sinica (Taipei, Taiwan).

### Computer analysis

Potential transcription factor-binding sites were mapped to the rat *Edn1* promoter using Match (http://gene-regulation.com/cgi-bin/pub/programs/match/bin/match.cgi).

### Transient transfection and luciferase activity assays

Cultured rat aortic SMCs were seeded at 3–5 × 10^5^ cells/well into 6-well plates. After cells had grown to approximately 80% confluence, previously prepared constructed plasmids were transfected using the jetPEI transfection reagent (Polyplus-transfection, New York, NY, USA). Luciferase activity was measured after cells had grown to confluence in DMEM for 24 h. Luciferase activities of the cell extracts were quantified with the Dual-Luciferase Reporter Assay System (Promega). Each sample was examined in triplicate in a minimum of three different experiments.

### Electrophoretic mobility shift assay and super-shift assay

Nuclear extracts of SHR and WKY VSMCs were prepared as previously described (Lee *et al.* 1988). The enhancer region probe was prepared from the complementary single-stranded DNA (sense: TTATGTGTGTTTTGCTTTATTCATGAAGACATGTTGTCA, antisense: TGACAACATGTCTTCATGAATAAAGCAAAACACACATAA) by melting at 95°C for 5 min followed by a cool-down phase of 3 h at ambient temperature. The probes were end-labeled with DIG-ddUTP and terminal transferase (Roche Applied Science). The binding reaction was carried out using a DIG Gel Shift Kit (Roche). The POU2F2 consensus probe and POU2F2 protein were from the super-shift assay kit. Antibodies against POU2F1, POU2F2, and CEBPB were purchased from Santa Cruz Biotechnology.

### Real-time quantitative (q)PCR and reverse-transcription (RT)-PCR

SHR and WKY VSMC total RNAs were extracted with the TRIzol reagent (Invitrogen). Tissue RNA was extracted with a Total RNA Mini Kit (Geneaid, New Taipei, Taiwan). For the qPCR, 0.5–1 μg of total RNA was taken for RT reaction, complementary (c)DNA was synthesized using SuperScript reverse transcriptase under priming of a random hexamer (Invitrogen), and gene expression was examined in a Bio-Rad iCycler optical system using the iQ™ SYBR green real-time PCR kit (Bio-Rad Laboratories). Data were normalized to α-actin reference. Primers used included forward primers *Pou2f2*: TTCATCCTCCTCCTCCTCCT; *Cebpb*: GACAAGCTGAGCGACGAGTA; *Edn1*: ACCACAGACCAAGGGAACAG; and *Acta2*: CACTTCCACAGAGCCAGACA and reverse primers *Pou2f2*: CTCCTTCGTCACTCCTGCTC; *Cebpb*: GACAGCTGCTCCACCTTCTT; *Edn1*: GGTCTTGATGCTGTTGCTGA; and *Acta2*: ATGGTGGTTTGGCTGAAGTC. For the RT-PCR, cDNA was synthesized using MMLV reverse transcriptase under priming of a random hexamer (BD Biosciences, San Jose, CA, USA), and the PCR used Taq DNA polymerase (Viogene, Taipei, Taiwan). Primers used included *Pou1f1*, forward, GATGGCAGGCACTTTAACCCCTTG, and reverse, GCAGATAAGACTTGCCTGTGGTAG. Primers are added to the reaction mixture and subjected to 30 cycles of amplification in a PCR machine (GeneAmp PCR System 2400; Applied Biosystems) at an annealing temperature of 55°C.

### Western blotting

For the extraction of cell nuclei and cytoplasmic proteins, first, use the cytoplasmic separation solution to suspend the cell pellet and lyse the cell membrane with a cell grinder, and then centrifuge at 1200 ***g*** for 10 min to obtain the cytoplasmic protein suspension and cell nucleus pellet. After the nucleus is precipitated by suspending it in the nucleus extraction solution, sodium chloride is added and centrifuged to obtain the nucleus protein. Nuclear proteins extracted from SHR and WKY VSMCs were probed with rabbit polyclonal anti-POU2F1 and anti-POU2F2 antibodies (1:500; Santa Cruz) or an anti-CEBPB antibody (1:250; Santa Cruz). The internal control was probed with a mouse monoclonal anti-poly (ADP ribose) polymerase (PARP) antibody (1:2000; NeoMarkers, Fremont, CA, USA). The primary antibody was then hybridized with horseradish peroxidase-conjugated host-specific secondary antibody (1:5000; Santa Cruz).

To detect the expression vectors, WKY VSMCs were cultured in six-well plates and transfected with the expression vector. The whole-cell lysate was obtained using a protease inhibitor (Roche)-contained RIPA Lysis Buffer (Merck) to suspend the cell pellet, place it on ice for 10 min, and centrifuge (13,000 ***g*** for 10 min) to collect the supernatant. The total protein extract was collected after cells had grown to confluence in DMEM for 24 h and probed with an anti-Myc antibody (1:2000; Life) after electrophoresis. Each electrophoretic analysis experiment uses an equal amount of total protein in the range of 10–30 μg.

### Measurements of ET1 release

Release of ET1 into the medium was determined for WKY VSMCs. After transfection of the *POU2F2* and *Cebpb* expression vectors, the culture medium was changed to serum-free medium. Supernatants were collected 6 h after changing the culture medium. Levels of EDN1 in the supernatants were measured with a human EDN1 TiterZyme Enzyme Immunometric Assay kit (Assay Design, Ann Arbor, MI, USA).

### Lentiviral production and transduction

VSV-G-pseudotyped lentiviruses were produced by co-transfecting TE671 cells with the sh*Pou2f2* or sh*Cebpb* clone, as well as two packaging plasmids: pMD.G and pCMVΔR8.91. Infectious lentiviruses were harvested at 12, 24, 48, and 72 h after transfection and were concentrated by ultracentrifugation (17,000 ***g*** for 3 h). SHR VSMCs were plated at 5 × 10^5^ cells/dish in 10-cm dishes and transiently transduced with lentivirus. After cells were infected with 100 µL of lentivirus for 24 h, the medium was replaced with fresh medium containing 1 µg/mL puromycin (Sigma-Aldrich) for drug-resistant cell selection. Cells were harvested after 48 h of transduction for subsequent analyses.

### Statistical analysis

All results are expressed as mean ± s.e.m. with *n* ≥3. Data were analyzed using Student's *t*-test for unpaired samples. Statistical significance was accepted at a value of *P* < 0.05. The results presented were derived from at least three separate experiments.

## Results

### Enhancer region of the *Edn1* promoter in SHR VSMCs

**Figure 1:**
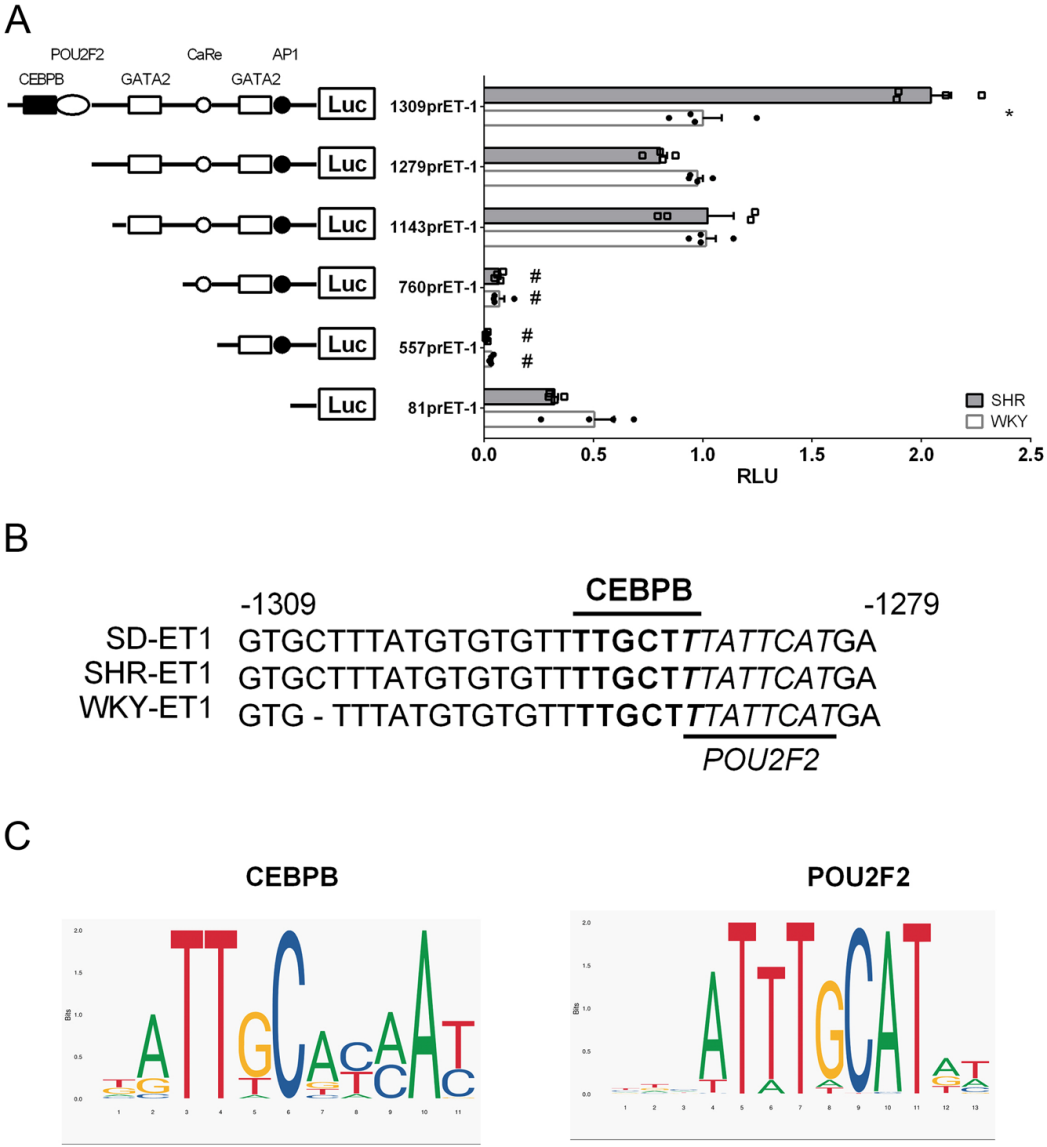
Analysis of the enhancer region of the rat endothelin promoter in vascular smooth muscle cells (VSMCs). (A) The region −1309/+0 and deletion at 5' DNA of rat *endothelin* (*ET*)*-1* were cloned in the pGL3-enhancer *Firefly* luciferase reporter vector and co-transfected with a pRL-CMV-derived *Renilla* luciferase reporter plasmid in spontaneously hypertensive rat (SHR) and Wistar-Kyoto (WKY) VSMCs. Transcriptional activity was normalized to the level of *Renilla* activity and expressed as fold activity of 1309prET1 in WKY rats. Smooth muscle cells were collected from the aortas of three SHR or WKY rats, and each data set consists of three experiments. (B) Sequence alignment between −1309 to −1079 upstream of the *Edn1* promoter in Sprague–Dawley (SD) rats, SHRs, and WKY rats. The underlined sequence region is the putative binding site for POU2F2 and CEBPB. (C) Sequence logo of two major putative elements on the *Edn1* promoter enhancer region. These logos were compiled by JASPAR software from published human cellular transcription factor-binding sites. Statistical data are shown as the mean ± s.e.m., *P < 0.05, ^##^*P* < 0.01. Student's *t*-test from three independent experiments. *vs 1309prET1 in WKY, ^#^vs 81prET1 in WKY rats. A full color version of this figure is available at https://doi.org/10.1530/JME-22-0178.

The rat *Edn1* promoter has a TATA box and putative cis-elements such as AP-1 and GATA2 sequences, a POU1f1 consensus sequence, and calcium response elements (upstream region of rat *Edn1* gene; Ensembl accession no. ENSRNOG00000014361) (Paul *et al.* 1995). To examine the enhancer region on the *Edn1* promoter in SHR VSMCs, we subcloned the −1309-bp *Edn1* promoter fragment into a reporter plasmid (1309prET1) and established five different 5'-end-deleted clones based on various cis-elements (1279prET1, 1143prET1, 760prET1, 557prET1, and 81prET1; Fig. 1A). The relative luciferase activity of the 1309 prET1 construct was 2.5-fold higher in 4-week-old SHR VSMCs than in WKY VSMCs, whereas the promoter activity of 1279prET1 and other shorter clones did not significantly differ between SHRs and WKY rats (Fig. 1A). This result shows that the *Edn1* promoter enhancer region was located at −1309 to −1279. Furthermore, when the −1143 to −760 region was removed from the ET1 promoter, 760prET1 promoter activity showed a 10-fold decrease in both SHR and WKY VSMCs. It was also lower than the 81prET1 clone containing the basal TATA box (Fig. 1A). This result shows that the −1143 to −760 region had VSMC-specific expression regulators, and the −557 to −81 region contained inhibitory effects.

To rule out polymorphisms in the ET1 promoter sequence affecting the promoter activity of different rat strains, we compared the 5' flanking sequences of the ET1 promoter from −1309 to −1279 among SD, SHR, and WKY rats. The ET1 promoter sequence was 99% identical in these rat strains (Fig. 1B). This confirmed a specific enhancer region of the *Edn1* promoter for transcriptional regulation of SHR VSMCs.

**Figure 2:**
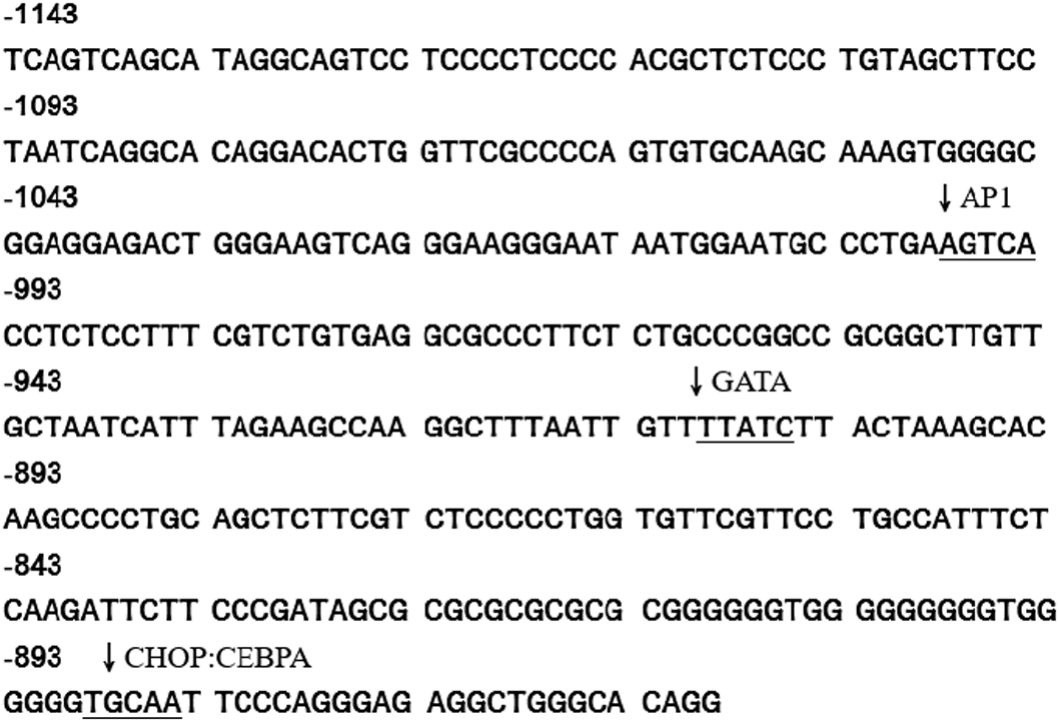
Sequence analysis of −1143 to −760 fragment on rat *Edn1* promoter. Arrows indicate the consensus sequences of the transcription factors GATA2, AP-1, and the CHOP:CEBPA complex.

In addition, the *Edn1* promoter activity was almost completely lost after deletion of the −1143 to −760 fragment in both SHR and WKY VSMCs (Fig. 1A). This shows that the main regulatory element of the endothelin promoter is contained between −1143 and −760. Sequence analysis revealed consensus sequences for three cis-acting elements, such as AP-1 (−998 to −994), GATA2 (−910 to −905), and CHOP:CEBPA (−789 to −785) (Fig. 2). GATA2 and AP-1 are also essential elements of human *EDN1* promoter activity (Lee *et al.* 1991, Kawana *et al.* 1995, Stow *et al.* 2011).

### POU2F2 and CEBPB binding to the enhancer region of the *Edn1* promoter

**Figure 3:**
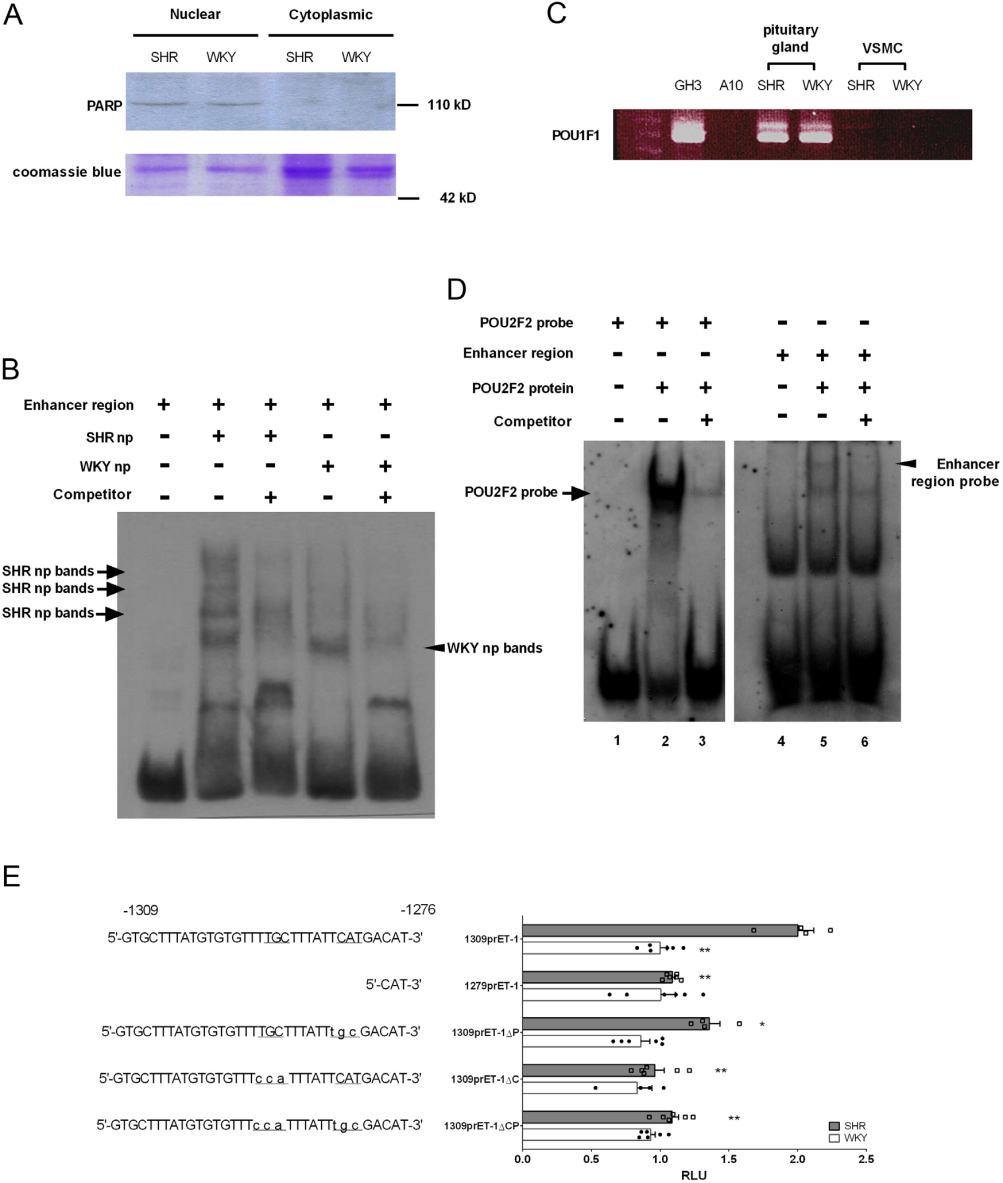
Different transcriptional regulation of the endothelin (ET)-1 promoter enhancer region. (A) Nuclear and cytoplasmic proteins were isolated from vascular smooth muscle cells (VSMCs) of spontaneously hypertensive rates (SHRs) and Wistar-Kyoto (WKY) rats. Western blot analysis of nuclear protein-poly(ADP ribose) polymerase (PARP) to confirm isolation purity. Coomassie blue staining of SDS-PAGE was used as an indicator of protein loading. (B) The DNA of the enhancer region was used as a probe and was incubated with SHR and WKY VSMC nuclear proteins for the EMSA analysis. Competing DNA was a 100-fold concentrated probe. Arrows indicate differences between probe-bound SHR and WKY VSMC nucleoproteins. (C) An RT-PCR analysis of the expressions of *Pou1f1* in a pituitary cell line (GH3), smooth muscle cell line (A10), pituitary gland (from SHRs and WKY rats), and VSMCs (from SHRs and WKY rats). (D) *Pou2f2* proteins were incubated with POU2F2 consensus sequence probes and enhancer region probes, and an EMSA was used to analyze protein–DNA interactions. Arrows indicate specific binding sites after mixing with competitor DNA of the POU2F2 consensus sequence. Arrowheads indicate specific binding sites after mixing with competitor DNA of the enhanced region sequence. (E) The POU2F2 and CEBPB consensus sequences of DNA sequences indicated on the bottom line were mutated in the 1309prET1 construct and co-transfected with Renilla reporter plasmids in SHR or WKY VSMCs. Statistical data are shown as the mean ± s.e.m., *P < 0.05, **P < 0.01. Student's *t*-test from three independent experiments. Smooth muscle cells were collected from the aortas of three SHR or WKY rats, and each data set consists of three experiments. *vs 1309prET1 in SHR rats. A full color version of this figure is available at https://doi.org/10.1530/JME-22-0178.

Protein binding was analyzed using DNA fragments involving the enhancer region as probes to address whether the *Edn1* promoter enhancer region is a regulator of *Edn1* upregulation in SHR VSMCs. Nuclear and cytoplasmic proteins were isolated from SHR and WKY VSMCs, and the purity was detected using PARP (Fig. 3A). Electrophoretic mobility shift assay (EMSA) results showed that the enhanced region could bind to nuclear extract proteins of SHR VSMCs (Fig. 3B, lane 2) but not to WKY VSMCs (Fig. 3B, lane 4). These results suggest that SHR VSMCs are specifically regulated by the enhancer region of the *Edn1* promoter.

Paul and colleagues identified a POU1F1-binding site at −1289 to −1283 of the *Edn1* promoter, but we found that SMCs of SHRs and WKY rats, and the A10 SMC line did not express *Pou1f1* mRNA (Fig. 3C). This result indicated that POU1F1 is not the regulator of *Edn1* upregulation in SHR VSMCs. We used JASPAR (Castro-Mondragon *et al.* 2022) and PROMO (Farré *et al.* 2003, Messeguer, *et al.* 2002) software to analyze the potential transcription factor-binding sites on the enhancer region of the rat *Edn1* promoter. We found two candidate binding sites for CEBPB and POU2F2 in the enhancer region of the *Edn1* promoter (Fig. 1B and C). To determine whether the POU2F2 protein can bind to the enhancer region of the *Edn1* promoter, we analyzed the binding of the POU2F2 protein to the enhancer region probe by an EMSA analysis. The data showed that the POU2F2 protein can bind to the POU2F2 consensus sequence with a high binding affinity (Fig. 3D, lane 2), and it can also bind to the enhancer region probe but with a relatively lower binding affinity (Fig. 3D, lane 5). To confirm whether CEBPB- and POU2F2-binding sites regulate the upregulation of the *Edn1* promoter in SHR VSMCs, we created core binding site mutations of CEBPB (1309prET1ΔC; TTGCTT→TCCATT) and POU2F2 (1309prET1ΔP; CAT→TGC). The CEPBB (1309prET1ΔC) or POU2F2(1309prET1ΔP) binding site mutation could fully reduce the upregulation of *Edn1* promoter activity in SHR VSMCs. These results indicated that CEBPB and POU2F2 are involved in *Edn1* upregulation (Fig. 3E). To further detect the roles of CEBP and POU2F2 functions in *Edn1* regulation, we also generated a double mutation of CEBPB and POU2F2 core binding sites. The promoter activity of the double-site mutation showed the same level as the single-site mutations (Fig. 3E). These results suggest that CEBPB and POU2F2 are co-factors of *Edn1* upregulation in SHR VSMCs.

### CEBPB and POU2F2 bind to the *Edn1* promoter enhancer region

**Figure 4:**
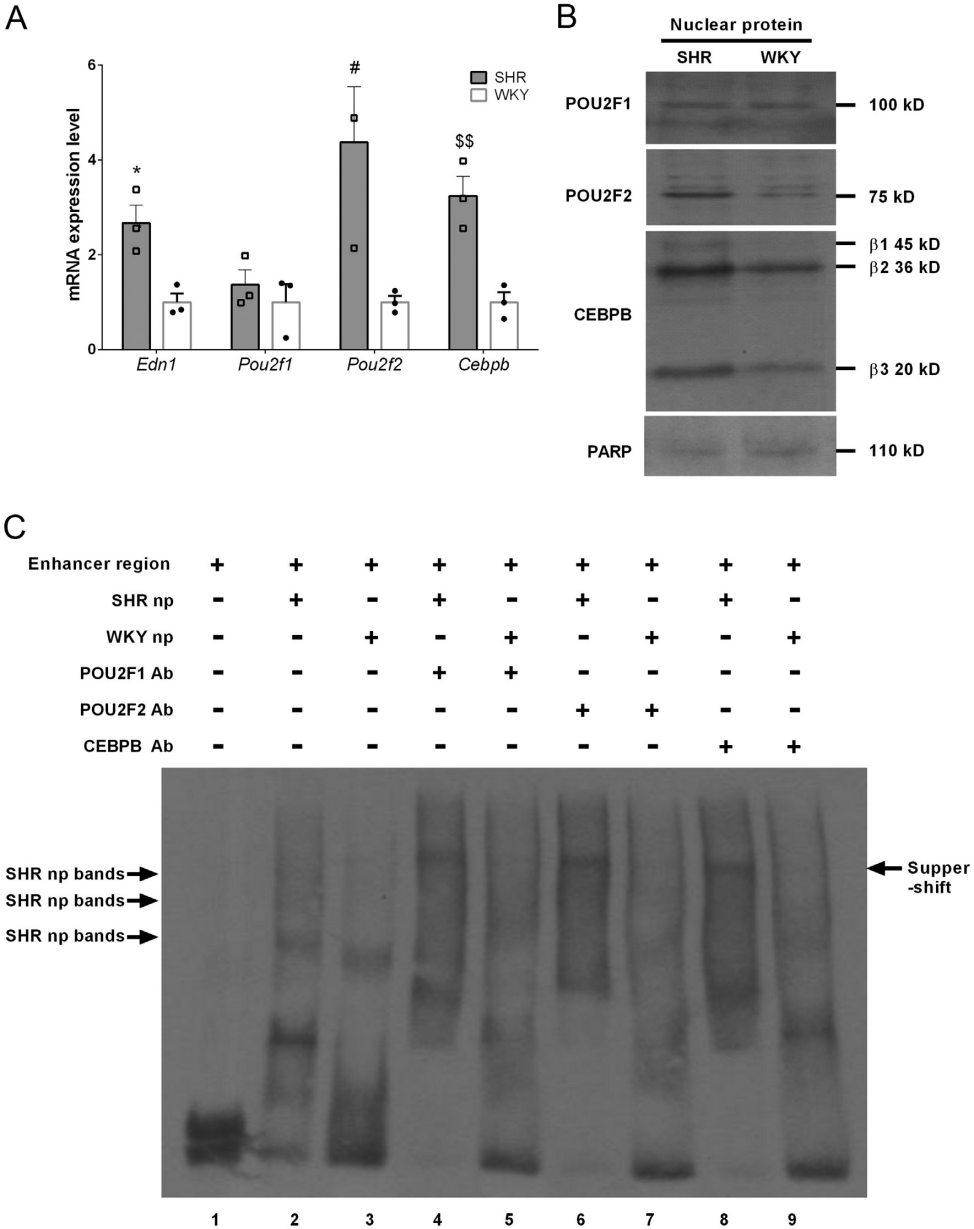
POU2F2 and CEBPB regulated endothelin (ET)-1 promoter activity in the enhancer region. (A) Real-time quantitative (q)PCR analysis of *Edn1*, *Pou2f1*, *Pou2f2*, and *Cebpb* expressions in the spontaneous hypertensive rat (SHR) and Wistar-Kyoto (WKY) rat vascular smooth muscle cells (VSMCs). Data are shown as the mean fold changes from three biological replicates relative to the control with s.e.m. error bars. *P < 0.05, ^#^*P* < 0.05, and ^$$^*P* < 0.01 vs gene expressions in WKY rats. (B) Western blot analysis of endogenous ET1, POU2f1, POU2f2, and CEBPB protein levels in nuclear extracts of SHR and WKY rat VSMCs. (C) Band super-shift analysis of endothelin promoter-enhancer region DNA fragment binding to POU2F1, POU2F2, and CEBPB proteins in SHR VSMCs. Lane 1, probe only. Lanes 2 and 3, nuclear protein extracted from SHR and WKY VSMCs, respectively, incubated with the Edn1 enhancer region. SHR or WKY nuclear proteins incubated with POU2F1 antibody (lanes 4 and 5), POU2F2 antibody (lanes 6 and 7), and CEBPB antibody (lanes 8 and 9). Arrow indicates a super-shifted band. Smooth muscle cells were collected from the aortas of three SHR or WKY rats, and each data set consists of three experiments. A full color version of this figure is available at https://doi.org/10.1530/JME-22-0178.

Previous studies showed that POU2F1 proteins share the same consensus sequence with POU2F2 proteins (Andersen & Rosenfeld 2001). Hatada and colleagues also reported that POU2F1 and POU2F2 can interact with CEBPB to regulate downstream gene expressions (Hatada *et al.* 2000). Therefore, we investigated whether POU2F1, POU2F2, and CEBPB bind to the *Edn1* promoter enhancer region in SHR VSMCs. qPCR and western blot results showed that POU2f1 mRNA and proteins were expressed at the same level between SHRs and WKY rats (Fig. 4A and B). In particular, mRNA and protein levels of POU2F2 and CEBPB in SHR VSMCs were, respectively, 4- and 3-fold higher than those in WKY rats (Fig. 4A and B). In the super shift analyses, we found that POU2F1, POU2F2, and CEBPB antibodies bonded to the *Edn1* promoter enhancer region by SHR VSMC nuclear proteins (Fig. 4C; lanes 4, 6, and 8) but not WKY VSMCs (Fig. 4C; lanes 5, 7, and 9). These results suggest that protein complexes from SHR nucleoproteins can bind to enhancer regions and may contain POU2F1, POU2F2, and CEBPB. Given the same expression levels of POU2F1, SHR VSMCs expressed higher levels of POU2F2 and CEBPB in response to *Edn1* upregulation.

### POU2F2 and CEBPB mediate *Edn1* upregulation in VSMCs

**Figure 5:**
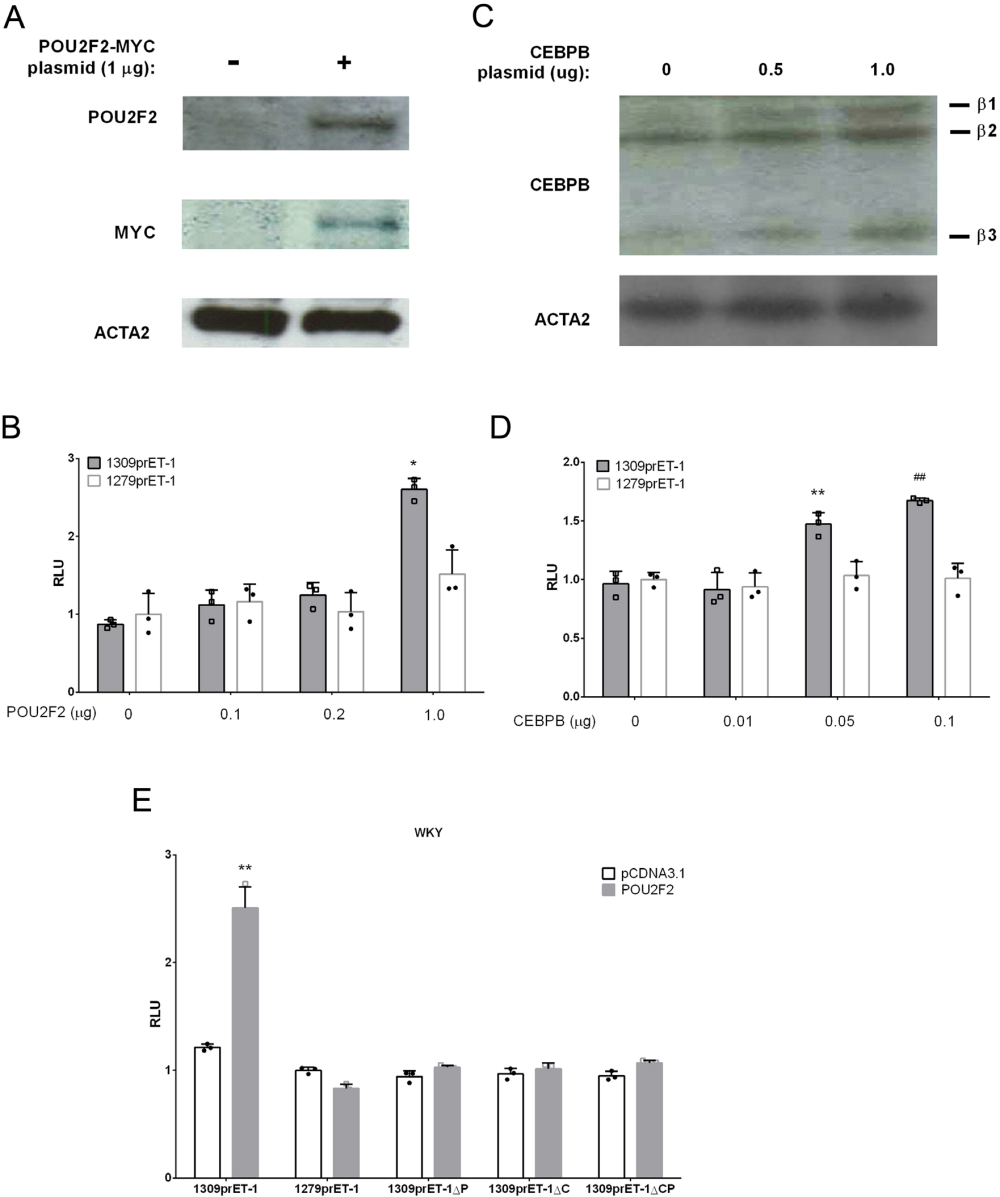
Overexpression of POU2F2 and CEBPB enhances endothelin (ET)-1 promoter activity in Wistar-Kyoto (WKY) vascular smooth muscle cells (VSMCs). (A) The POU2F2-myc expression vector was transfected into WKY VSMCs for 1 day. Western blotting shows POU2F2 and MYC overexpression in WKY VSMCs. (B) WKY VSMCs were co-transfected with different concentrations of the POU2F2 vector and two lengths of the *Edn1* promoter-firefly luciferase reporter vector (1309prET1 and 1279prET1). Promoter activity was assessed by relative firefly luciferase activity and *Renilla* luciferase activity. The 1279prET1 promoter activity without POU2F2 transfection was set to 1. Statistical data are shown as the mean ± s.e.m., Student's *t*-test from three independent experiments. *P < 0.05. *vs 1279prET1 with 1 μg POU2F2. (C) Various concentrations of the CEBPB vector were transfected into WKY VSMCs for 24 h. Expression levels of CEBPB were confirmed by a western blot analysis. (D) WKY VSMCs transfected with various concentrations of the CEPB vector were co-transfected with 1309prET1 and 1279prET1. 1279prET1 promoter activity without CEBPB transfection was set to 1. Statistical data are shown as the mean ± s.e.m., Student's *t*-test from three independent experiments. Smooth muscle cells were collected from the aortas of three SHR or WKY rats, and each data set consists of three experiments. **P < 0.01, ^##^ *P* < 0.01. *vs 1279prET1 with 0.05 μg CEBPB. ^##^vs 1279prET1 with 0.05 μg CEBPB. (E) POU2F2 expression vector (1 μg) co-transfected with various site-directed mutants of *Edn1* promoter constructs, such as 1309prET1ΔP, 1309prET1ΔC, and 1309prET1ΔCP. **P < 0.01. *vs 1279prET1 without POU2F2. A full color version of this figure is available at https://doi.org/10.1530/JME-22-0178.

To determine the regulatory mechanism of the overexpressed region, POU2F2 and CEBPB expression plasmids were co-transfected with the *Edn1* promoter into WKY VSMCs. Western blotting showed that cells transfected with the POU2f2 and CEBPB plasmids exhibited upregulated protein expression levels (Fig. 5A and C). Furthermore, POU2F2 increased 1309prET1 promoter activity by 2-fold but not 1279prET1 promoter activity in WKY VSMCs (Fig. 5B). CEBPB increased 1309prET1 promoter activity in WKY VSMCs by 1.5-fold but had no effect on 1279prET1 promoter activity (Fig. 5D). Therefore, we propose that POU2F2 may play a significant role in enhancing the *Edn1* promoter in SHR VSMCs. To further determine the regulation of the enhancer region of the *Edn1* promoter by POU2F2, we analyzed activities of various site-mutated *Edn1* promoter constructs, such as 1309prET1ΔP, 1309prET1ΔC, and 1309prET1ΔCP. We found that POU2F2 did not enhance POU2F2 core consensus-mutated *Edn1* promoter activity such as 1309prET1ΔP and 1309prET1ΔCP (Fig. 5E). POU2F2 overexpression also did not improve CEBPB core consensus-mutated *Edn1* promoter activity such as 1309prET1ΔC (Fig. 5E). These results suggest that POU2F2 is the main factor that enhances *Edn1* expression in SHR VSMCs. At the same time, CEBPB is a cofactor that promotes and activates *Edn1* promoter activity.

### POU2F2 and CEBPB regulate endogenous EDN1 expression

**Figure 6:**
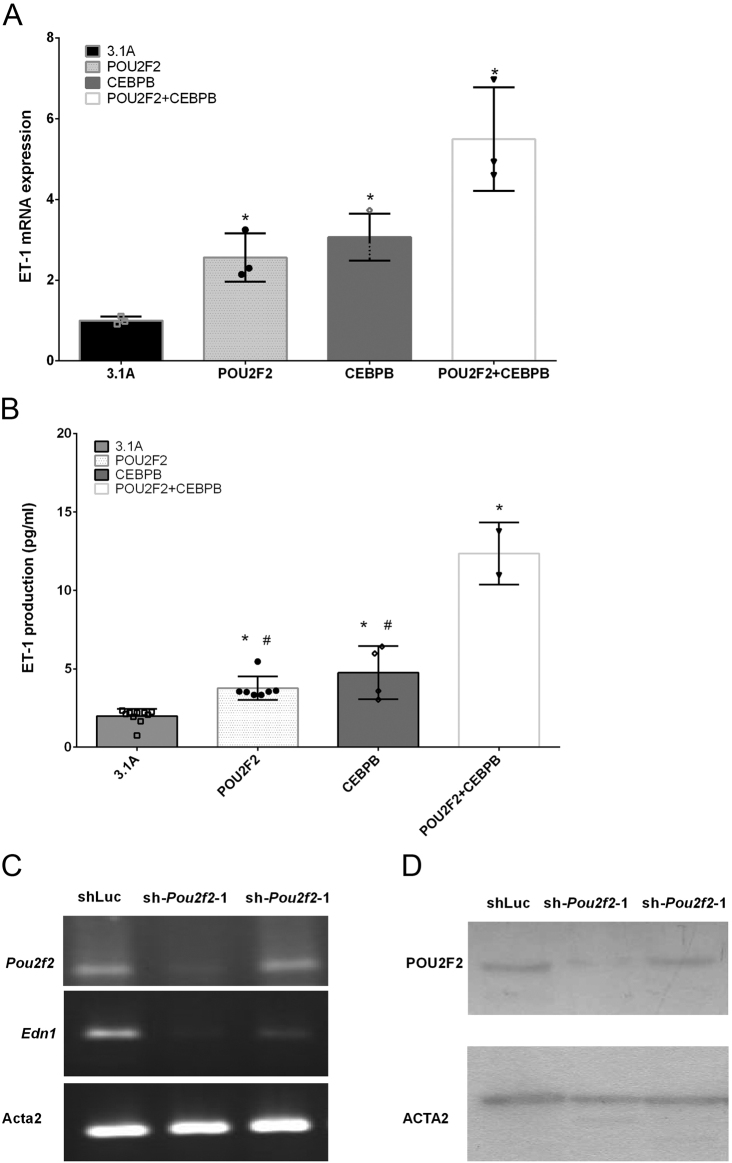
Overexpression of POU2F2 and CEBPB affects endogenous endothelin (ET)-1 expression. Total RNA or culture medium from Wistar-Kyoto (WKY) vascular smooth muscle cells (VSMCs) was collected from cells transfected with pcDNA3.1A (3.1A), POU2F2, CEBPB, and POU2F2 + CEBPB expression vectors. (A) *Edn1* mRNA expression was examined by a qPCR. Data shown were normalized to the expression of the *α-actin* reference gene, and the expression level with 3.1A empty vector transfection was set to 1. (B) EDN1 ELISA for measurement of EDN1 secretion in VSMC culture medium. *P < 0.01. *vs 3.1A empty vector transfection. (C and D) Cells were infected with lentivirus-expressing POU2f2 shRNAs (sh-*Pou2f2*-1 and sh-*Pou2f2*-2) for 24 h. As a negative control, cells were infected with a luciferase shRNA-containing lentivirus (sh-Luc). POU2F2 shRNA infection was confirmed by an RT-qPCR (C) and western blotting (D). Smooth muscle cells were collected from the aortas of three SHR or WKY rats, and each data set consists of three experiments.

To further characterize the effects of POU2F2 and CEBPB on endogenous EDN1 expression, we examined the endogenous EDN1 expression level after POU2F2 and CEBPB overexpression or inhibition. We found that POU2F2 or CEBPB overexpression was sufficient to induce EDN1 mRNA and ET1 secretion levels of WKY VSMCs (Fig. 6A and B). However, after co-transfection of the POU2F2 and CEBPB plasmids into WKY VSMCs, EDN1 mRNA and secretion levels were higher than those in cells transfected with POU2F2 or CEBPB alone (Fig. 6A and B). These data suggest that POU2F2 and CEBPB regulate EDN1 upregulation with an additive effect.

On the other hand, to study the effect of POU2F2 in EDN1 upregulation of SHR VSMCs, *Pou2f2* shRNA was used to suppress the endogenous *Pou2f2* gene expression in cells. Results show that sh*Pou2f2*-1 effectively reduced levels of *Pou2f2* mRNA and protein in SHR VSMCs (Fig. 6C and D). Figure 5C shows that sh*Pou2f2*-1 significantly reduced *Edn1* mRNA expression in SHR VSMCs, indicating that POU2F2 is required for the upregulation of EDN1 expression.

### *POU2F2* and *CEBPB* expression levels in dedifferentiated human VSMCs and enhancer region sequence conservation

**Figure 7:**
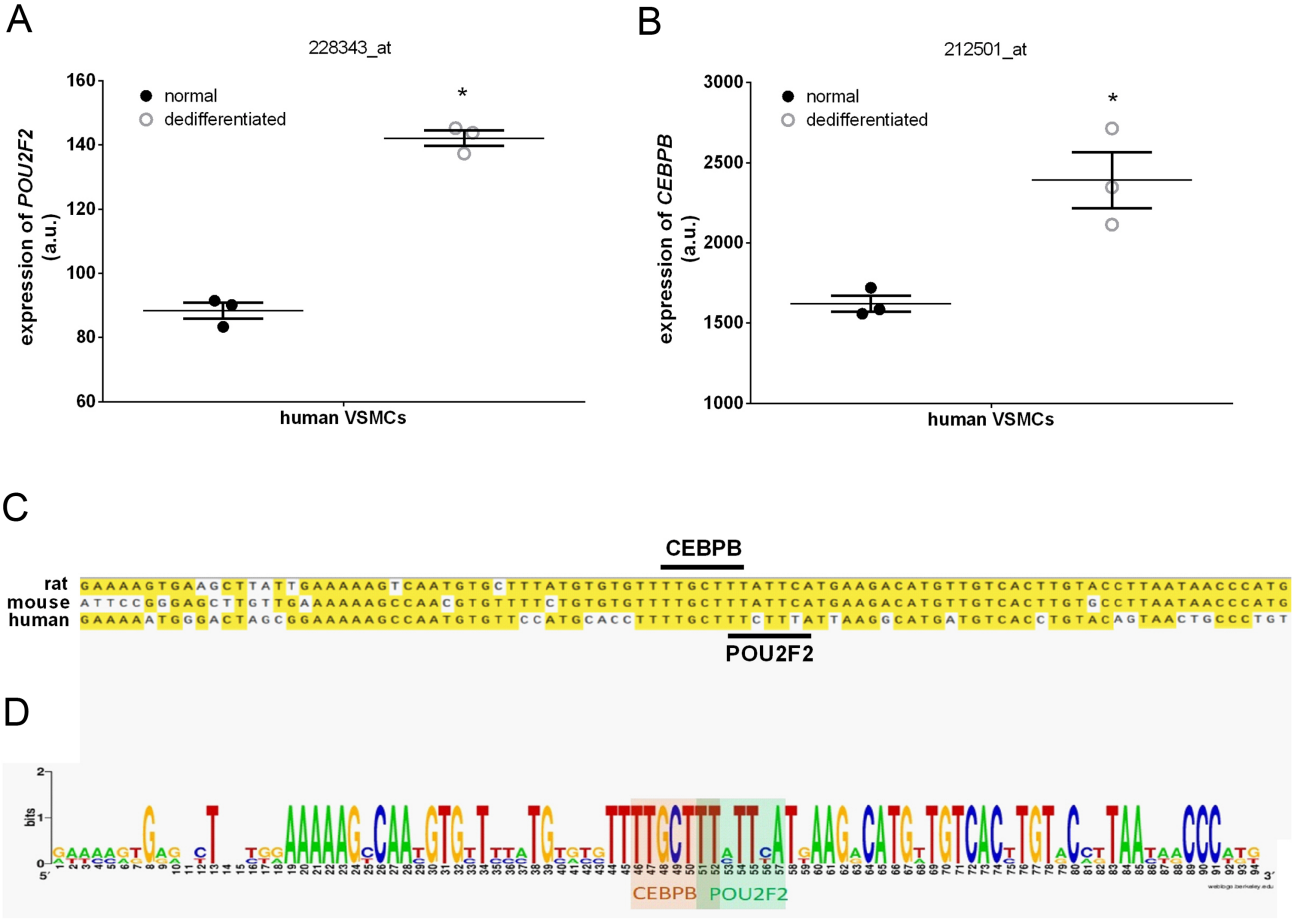
POU2F2 and CEBPB expressions increased in dedifferentiated human vascular smooth muscle cells (VSMCs). *POU2F2* and *CEBPB* mRNA expression values from a microarray dataset were obtained from NCBI's Gene Expression Omnibus repository. (A) The 228343_at probe set was used to detect human *POU2F2* mRNA expression. (B) The 212501_at probe set was used to detect human *CEBPB* mRNA expression *P < 0.05. (C) Sequence alignment of rat, mouse, and human endothelin (ET)-1 promoter enhancer regions. Underlined sequence regions are POU2F2 and CEBPB consensus sequence of *Edn1* promoters (https://doi.org/10.1530/JME-22-0178.

Dedifferentiation of VSMCs regulates vascular remodeling and contributes to the development of hypertension (Touyz *et al.* 2018). Expressions of *POU2F2* and *CEBPB* by dedifferentiated human VSMCs were examined using a microarray database from the Gene Expression Omnibus repository of the NCBI (dataset: GDS3851). The microarray datasets included normal human (h)VSMCs and dedifferentiated hVSMCs (Balint *et al.* 2015). The *POU2F2* probe set, 228343_s_at, and *CEBPB* probe set, 212501_at, were used to detect expression levels of human *POU2F2* (Fig. 7A) and *CEBPB* (Fig. 7B) mRNA. *POU2F2* and *CEBPB* expression levels were significantly higher in dedifferentiated hVSMCs compared to undifferentiated hVSMCs. In addition, a significant 1.52-fold upregulation in the CEBPB expression level was also confirmed in the adrenal glands of hypertensive mice (Friese *et al.* 2005).

To understand interspecific differences in *EDN1* expression affected by POU2F2 and CEPBP, we used Clustal Omega software to perform multiple sequence alignments of the distal regions of the rat (NM_012548), mouse (NM_010104), and human (NM_001955) EDN1 promoters (Fig. 7C). Results show that the 5' flanking regions of the human and mouse *EDN1* genes have a highly conserved sequence with the rat *Edn1* promoter −1309 to −1279 region. Web logo software showed that this region has a consensus sequence for POU2F2 and CEBPB (Fig. 7D).

## Discussion

**Figure 8:**
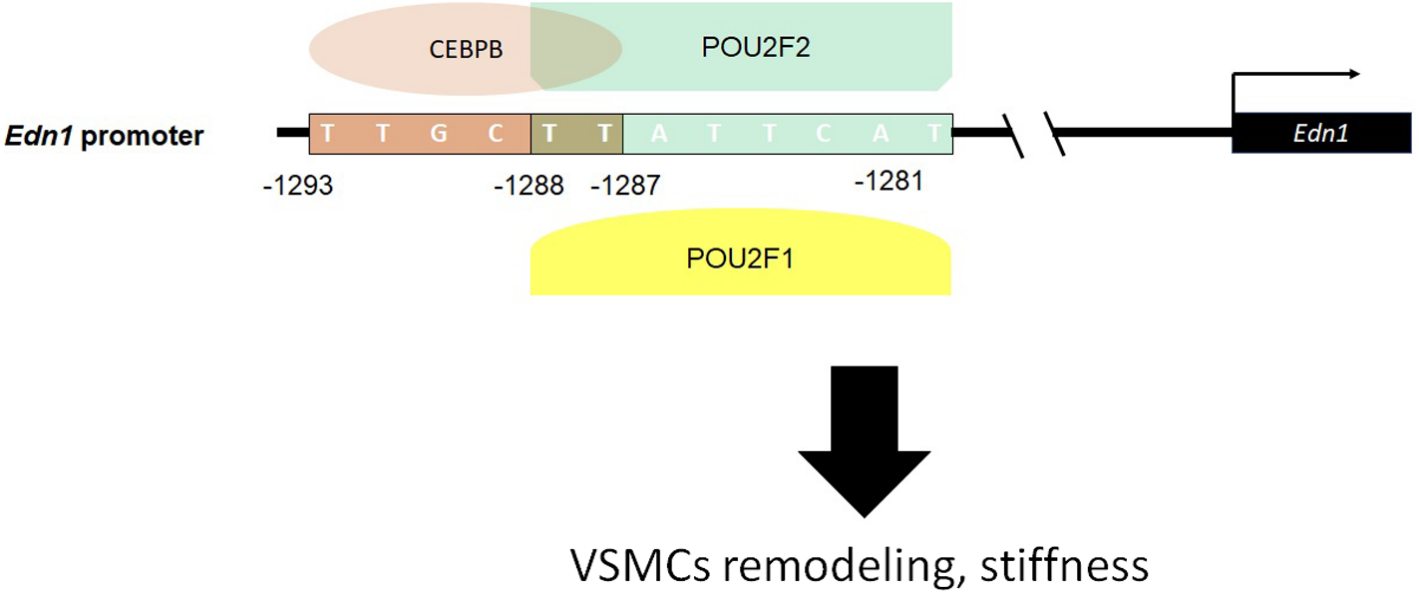
Hypotheses of the molecular mechanism for the cooperation of POU2F2 and CEBPB in the spontaneous hypertensive rat (SHR) vascular smooth muscle cells (VSMCs) to enhance endothelin (ET)-1 expression. The enhancer region on the Edn1 promoter enhanced *Edn1* gene expression in SHR VSMCs. The *Edn1* promoter enhancer region binds with different protein complexes between SHR and Wistar-Kyoto (WKY) VSMCs. In SHR VSMCs, the protein complexes included POU2F1, POU2F2, and CEBPB, but not in WKY VSMCs. Especially for EDN1 overexpression, POU2F2 and CEBPB played essential roles in promoter regulation. A full color version of this figure is available at https://doi.org/10.1530/JME-22-0178.

To characterize the molecular mechanisms involved in the overexpression of EDN1 in SHR VSMCs, we constructed a mutated enhancer region of the *Edn1* promoter and manipulated expressions of POU2F2 and CEBPB. Our results showed that increased POU2F2 and CEBPB led to enhanced *Edn1* promoter activity and EDN1 expression by WKY VSMCs. In contrast, the downregulation of POU2F2 and CEBPB suppressed EDN1 promoter activity and expression by SHR VSMCs. Figure 8 summarizes the molecular mechanism that seems to occur with ET1 overexpression by SHR VSMCs. The rat *Edn1* promoter enhancer region is located at −1281 to −1293 upstream of the transcription start point and binds to the POU2F1, POU2F2, and CEBPB protein complex. Protein expression levels of POU2F2 and CEBPB were upregulated in SHR VSMCs, resulting in enhanced EDN1 expression levels. Although POU2F1 was present in this protein complex, POU2F1 protein expression levels did not change between SHRs and WKY rats. This suggests that POU2F1 is not an important regulator of *Edn1* promoter activity. The POU2F2-binding site in the enhancer region of the rat *Edn1* promoter is not a typical POU2F2 consensus sequence. It can only produce relatively low protein–DNA interactions, which may explain the additive effect of CEBPB and POU2F2 in WKY VSMCs. CEBPB and POU2F2 regulate the expression of EDN1 in SHR VSMCs and may affect vascular remodeling and stiffness for a long time, resulting in a gradual increase in BP. Our results support a novel regulatory role of the *Edn1* promoter enhancer region in SHR VSMCs. Our results support a novel molecular mechanism of POU2F2 and CEBPB in regulating EDN1 expression. Co-expression of POU2F2 and CEBPB induced higher levels of EDN1 expression.

Rat and human *EDN1* promoters share up to 85% sequence similarity (Paul *et al.* 1995) and thus may have similar transcriptional regulation. The GATA2 consensus sequence between −148 and −117 upstream of the human endothelin promoter and the AP-1 consensus sequence between −117 and −98 are the necessary regulatory elements for the expression of endothelin in human ECs (Lee *et al.* 1991, Kawana *et al.* 1995, Stow *et al.* 2011). We found that the deletion of the endothelin promoter −1143 to −760 fragments would lead to a complete loss of promoter activity (Fig. 1A), and the consensus sequences of GATA2 and AP-1 were also contained between these fragments. This result suggests that GATA2 and AP-1 may also be required for basal transcription of edn1 in cultured VSMCs.

Although EDN1 expression levels in plasma were not significantly elevated in SHRs, plasma levels of EDN1 were also inconsistent in essential hypertensive patients (Schiffrin *et al.* 2001, Kostov *et al.* 2021). These differences may be due to the half-life of EDN1 in plasma of only 1–2 min (Dhaun *et al.* 2008) and the release of 80% endothelin via the albumin side of ECs (Gao *et al.* 2016). Therefore, without salt stimulation or other pathological stress, the plasma EDN1 concentration in SHRs cannot reflect the true level. On the other hand, elevated local EDN1 levels were found in vessel walls of hypertensive patients, and the higher growth rate of SHR VSMCs could be inhibited by EDNRA and beta-blockers (Hamada *et al.* 1990, Barton & Yanagisawa 2008, Kostov *et al.* 2021).

Previous studies indicated that the rat *Edn1* promoter enhancer region sequence is a POU1F1 consensus sequence (Paul *et al.* 1995, Andersen & Rosenfeld 2001). POU1F1, a pituitary-specific transcription factor, is a member of the POU-domain protein family (also named POU1F1) (Andersen & Rosenfeld 2001). POU1f1 is expressed by the anterior pituitary gland and was not expressed by SHR VSMCs (Fig. 3C). There are two DNA-binding domains, POUs and POUh, among POU-domain family proteins (Wegner *et al.* 1993). The POUs domain specifically binds to the NNCAT sequence, while the POUh domain generally binds to A/T-rich sequences with a TAAT core (Rosenfeld 1991). It was reported that the POU2F1 POU-domain can interact with other POU-domain family proteins such as POU1F1, POU2F1, POU2F2, and POU3F1. These interactions can form homomeric or heteromeric complexes to regulate the immunoglobulin heavy chain promoter (Verrijzer *et al.* 1992). Our EMSA indicated that POU2F2 could bind to the POU1F1 consensus sequence with a lower affinity than to the POU2F2 consensus sequence (Fig. 3D). The super-shift assay indicated that the nuclear binding protein from SHR VSMCs for the POU1F1 consensus sequence may contain POU2F1, POU2F2, and CEBPB (Fig. 4C).

POU domain family proteins can not only interact with but also recruit DNA-binding proteins as co-activators or co-repressors to regulate target genes that depend on their specific DNA-binding sequences (Andersen & Rosenfeld 2001). Previous studies showed that the interaction between POU2F1 and POU2F2-OCA-B requires an ‘A’ at position 5 of the octamer site (ATGCAAAT), whereas POU2F1–VP16 interactions require the GARAT part of the TAATGARAT site to form a ternary complex (Babb *et al.* 1997). In addition, sequences outside the POU domain octamer-binding motif can recruit other specific coregulators and provide specific biological functions (Andersen and Rosenfeld 2001). In the IL8 promoter, the POU domain-binding site overlaps with the C/EBP consensus sequence, and POU2F1 interacts with C/EBPB to inhibit the expression activity of IL8 (Wu *et al.* 1997). We identified a POU domain octamer sequence that overlapped the C/EBP consensus sequence on the *Edn1* promoter enhancer region, and POU2F1, POU2F2, and CEBPB were all found to bind to the enhancer fragment and regulate *Edn1* promoter activity.

CEBPB was found in the nucleus during the early stage of differentiation of coronary smooth muscles from proepicardial cells, and it facilitates 10T1/2 fibroblast differentiation into SMCs (Chang *et al.* 2003). Lauth and colleagues reported that EDN1 expression also decreased after inhibiting the activity of CEBPB in vascular ECs (Lauth *et al.* 2000, Kelkenberg *et al.* 2002). Yamashita *et al.* also showed that the CAAT box only affected POU1F1-enhanced EDN1 expression but not basal transcriptional activity in ECs under hypoxic conditions (Yamashita *et al.* 2001). This result suggests that the role of the CAAT box may be as an enhancer of EDN1 expression. It is known that the CAAT box is not only a single type of transcription factor but is included in the CAAT transcription factor, nuclear factor-Y, CCAAT displacement protein, and CCAAT/enhancer-binding protein (CEBP) (Mantovani *et al.* 1998). In SHRs and BPH mice, CEBPB expression was significantly upregulated compared to the normal control group. We also found higher expression levels of *POU2F2* and *CEBPB* in dedifferentiated human VSMCs (Fig. 7). In this context, the additive effect of POU2F2 and CEBPB may be able to explain the progressive increase in BP during the development of hypertension. In conclusion, this discovery provides a deeper understanding of the molecular regulation mechanism of hypertension and identifies novel directions for the development of new clinical treatments for hypertension. CEBPB and POU2F2 expression in vascular SMCs may also serve as prognostic biomarkers.

Estrogen (such as 17beta-estradiol, E2) represses *Edn1* gene expression in many cell types, including ECs (Akishita *et al.* 1998), aortic VSMCs (Akishita *et al.* 1996, Hong *et al.* 2004), and cardiac fibroblasts (Chao *et al.* 2005). E2 may regulate the expression of the *Edn1* gene by inhibiting AP-1 activity (Juan *et al.* 2004). The −1143 to −760 fragment of the endothelin promoter is the determining region for the main activity of rat *Edn1*, and a consensus sequence of AP-1 was also found in this region (Fig. 1). Therefore, the AP-1 consensus sequence on this fragment may be involved in the regulation of EDN1 expression by estrogen.

## Declaration of interest

The authors declare that they have no known competing financial interests or personal relationships that could have appeared to influence the work reported in this paper.

## Funding

This study was supported by the grants from Taipei Medical University (TMU110-AE1-B24) to Tien-Chun Yang.

## Author contribution statement

Tien-Chun Yang: Conceptualization, Methodology, Formal analysis, Writing – review & editing, Funding acquisition. Mei-Hua Lu: Conceptualization, Methodology, Investigation. Wei-Jie Wang: Investigation, Writing – original draft. Jang-Yi Chen: Conceptualization, Methodology, Formal analysis, Writing – review & editing.
